# Alcohol Poisoning

**Published:** 1887-07

**Authors:** W. B. Parks

**Affiliations:** Atlanta, Ga.


### ALCOHOL POISONING.*

BY W. B. PARKS, M. D., ATLANTA, GA.

The origin of this remarkable agent, together with its physical and chemical characters, are so well known that any allusion to them would be quite superfluous. We will consider its influence upon the animal economy as a narcotic and pass on.

*Read before the Atlanta Society of Medicine.

Alcohol and alcoholic liquors generally, when taken into the stomach in moderate quantities, invariably produce a greater or less degree of excitement—that is, they augment the temperature of the body, accelerate the movements of the heart and respiratory organs, give tone and strength to the muscles, energy and rapidity to the mind, and in general call up a train of pleasurable emotions.

When the limits of moderation are transgressed, and a quantity sufficient to produce intoxication or drunkenness is taken, the consequences are materially different from that of the first excitement. This first excitement soon puts on a more formidable character, being attended by difficult articulation, giddiness, incoherency of the mind, and very frequently true delirium; drowsiness next succeeds, which terminates in a deep and sometimes profound sleep from which the individual awakes with headache and very often nausea and vomiting. The results are sometimes different ; the somnolency just described passes not into sleep, but a state of general insensibility, accompanied by feeble and moderately rapid pulse, stertorous breathing and a dilated pupil.

It more frequently occurs that life is destroyed, either by the occurrence during the fit of intoxication of apoplexy, in an individual predisposed to the disease, or in consequence of his exposure to extreme cold or accidental suffocation during this lethargic or somnolent state. Should the spirituous liquors be drank, not in divided portions, but in large quantity, at one draught, apoplexy or coma, with stertorous breathing, sets in almost immediately, and death follows usually within a few hours.

Looking to the preceding history of symptoms, alcohol, it would appear, should be classed with the purely narcotic poisons. Cases occur in which, after the cessation of the narcotic action, symptoms of gastro-intestinal irritation and inflammation have set in which justifies the position usually assigned to it in the arrangement of toxicologists. The morbid appearances which have been observed in the stomach in cases of alcohol poisoning are usually redness and vascularity of its mucous linings. Such appearances are, according to Orfilla, invariably witnessed in the stomachs of the lower animals. In man, though sometimes found, they are generally wanting.

In the head, as might have been anticipated from the symptoms, the principal lesions are to be found. Now, in actual alcohol poisoning in man, we find venous and capillary congestion, serous effusions into the ventricles and between the membranes and extravasations of blood into the substance of the brain, producing apoplexy. When delirium tremens proves fatal, serous effusion is found in the brain. If death should take place at once, after a large quantity of alcohol had been swallowed, *post mortem* examination would not show any lesion of the brain or any organ. When taken in small quantities for quite a while, it sets up a disease of the brain or an anæmic condition of the brain. As we have before stated, it accelerates the circulation, forcing the blood to the brain, thereby over-distending the blood-vessels of the brain. You will remember that, anatomically, the most of the blood-vessels of the brain, especially the veins, have not the same number of coats as the blood-vessels in any other portion of the body. With this anatomical fact, you can see at once that when the blood-vessel has been distended, and when the stimulant is withdrawn, it leaves the blood-vessels debilitated, yet they are called on to receive the blood, that the brain may be nourished, and in every round of circulation this once over-distended blood-vessel refuses to receive and convey the blood normally. Thence we have an anæmic brain, to say nothing of effusion into brain substance, for this class occurs only in large doses. We have in this condition the reason that once a man contracts the habit of taking his dram, as he would say, "in moderation," he cannot reform; a disease is set up in the brain; the appetite is in the brain, not in the stomach.

I have made it a point to get men who drink fermented liquors, as a beverage to give me their experience, as to its affects on them and I find all give me the same history in regard to the appetite. Many drink whisky holding their noses, the taste is so disgusting, yet an anæmic brain calls for it. The man drinks the poison though he knows it will kill him. He would rather die with this depressed feeling relieved by drinking than die with a brain perishing for want of food, for the blood-vessels, debilitated by a former stimulant, cannot receive and carry the blood normally.

Conclusion: You may organize the world into temperance societies; you may send up petitions from every State in the Union to the legislatures praying to regulate the indiscriminate sale of spirituous liquors; you may tell and show the poor inebriate that he is on the brink of the drunkard's grave, yet if you do not relieve his anæmic brain, if you do not cure the disease set up in the brain by this poisonous agent, he will cry, "Give me drink, or I die!"

